# Supplementary material for: AlphaFold2 models of the active form of all 437 catalytically competent human protein kinase domains
Source: bioRxiv. 2023 Sep 3:2023.07.21.550125. Originally published 2023 Jul 25. Preprint. [Version 2] doi: 10.1101/2023.07.21.550125 (PMC10401967; doi:10.1101/2023.07.21.550125)
Supplement: 1 [file NIHPP2023.07.21.550125V2-supplement-1.pdf]

bioRxiv preprint doi: https://doi.org/10.1101/2023.07.21.550125; this version posted September 3, 2023. The copyright holder for this preprint (which was not certified by peer review) is the author/funder, who has granted bioRxiv a license to display the preprint in perpetuity. It is made available under aCC-BY 4.0 International license.

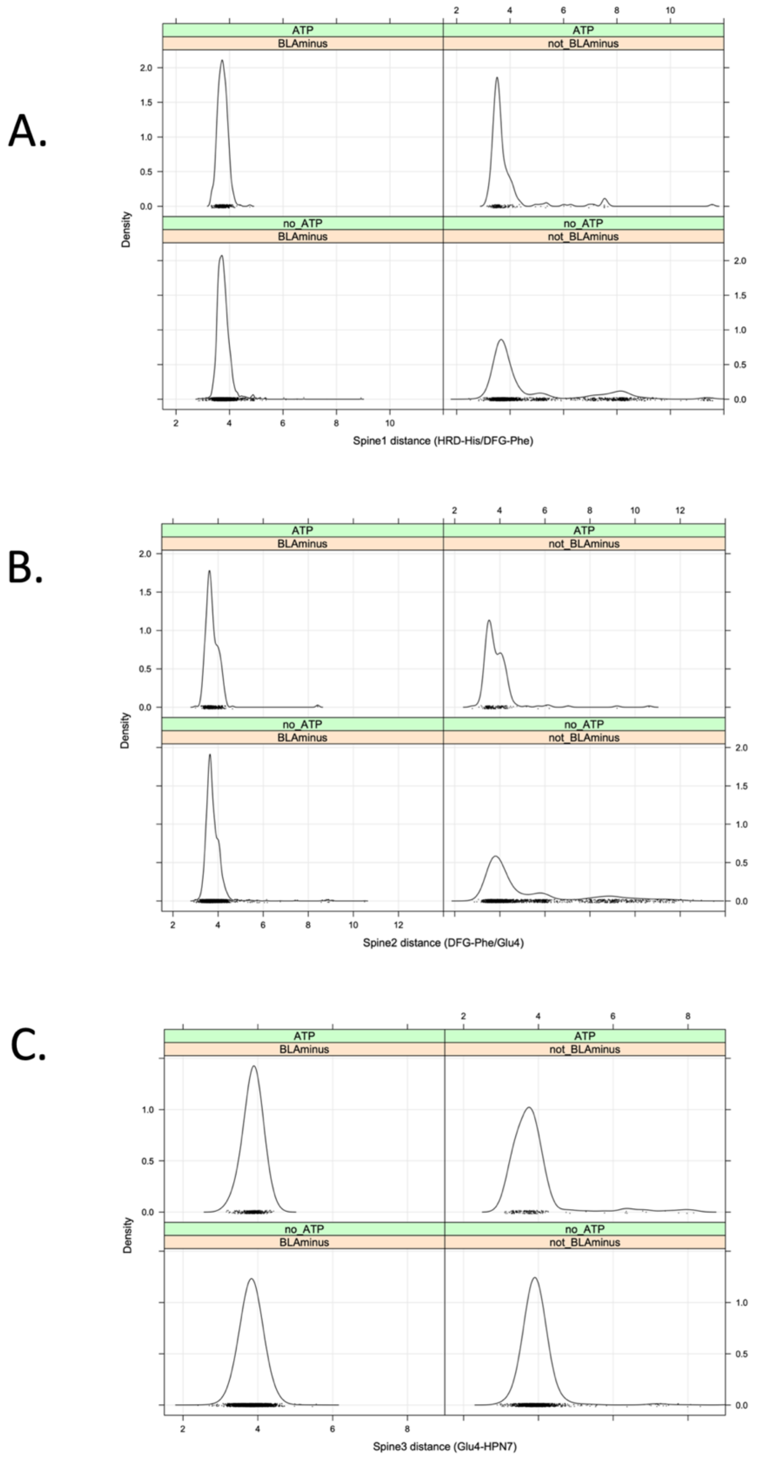

Supplementary Figure 1. Distribution of the Spine1, Spine2, and Spine 3 distances. The Spine distances are defined as the closest distance among all side-chain atom pairs between the two residues.

bioRxiv preprint doi: https://doi.org/10.1101/2023.07.21.550125; this version posted September 3, 2023. The copyright holder for this preprint (which was not certified by peer review) is the author/funder, who has granted bioRxiv a license to display the preprint in perpetuity. It is made available under aCC-BY 4.0 International license.

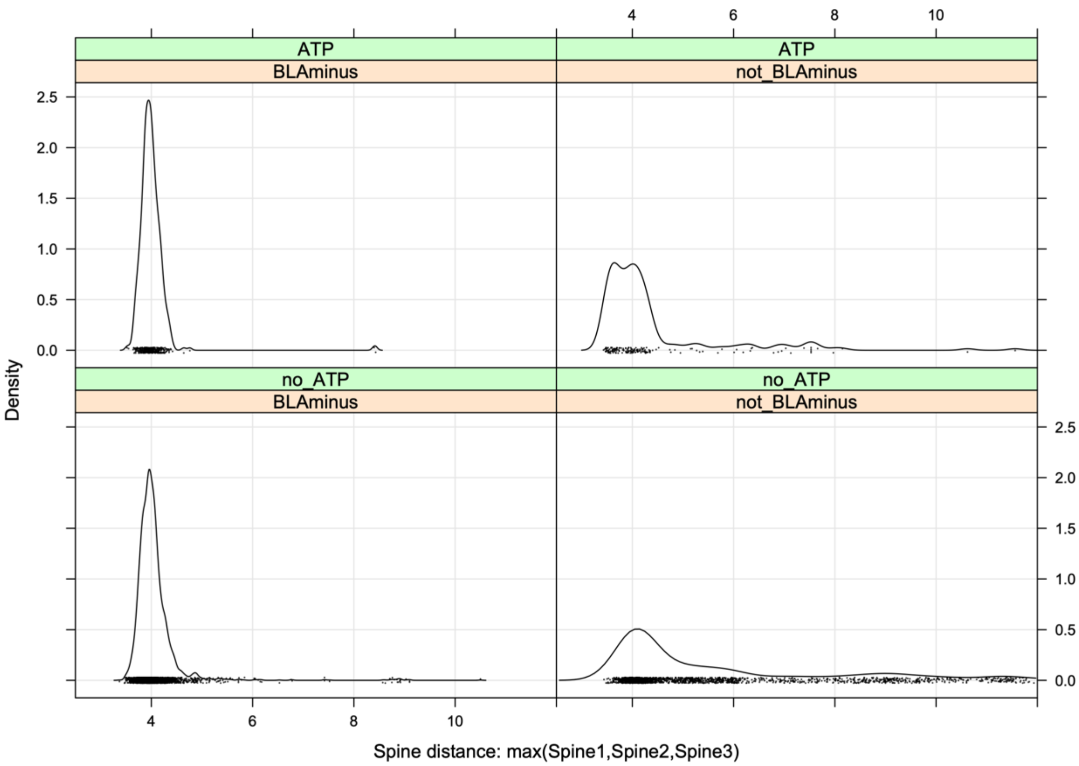

Supplementary Figure 2. Distribution of the Spine distance, which is the maximum of Spine1, Spine2, Spine3 (Supplementary Figure 1) for each kinase structure.

bioRxiv preprint doi: https://doi.org/10.1101/2023.07.21.550125; this version posted September 3, 2023. The copyright holder for this preprint (which was not certified by peer review) is the author/funder, who has granted bioRxiv a license to display the preprint in perpetuity. It is made available under aCC-BY 4.0 International license.

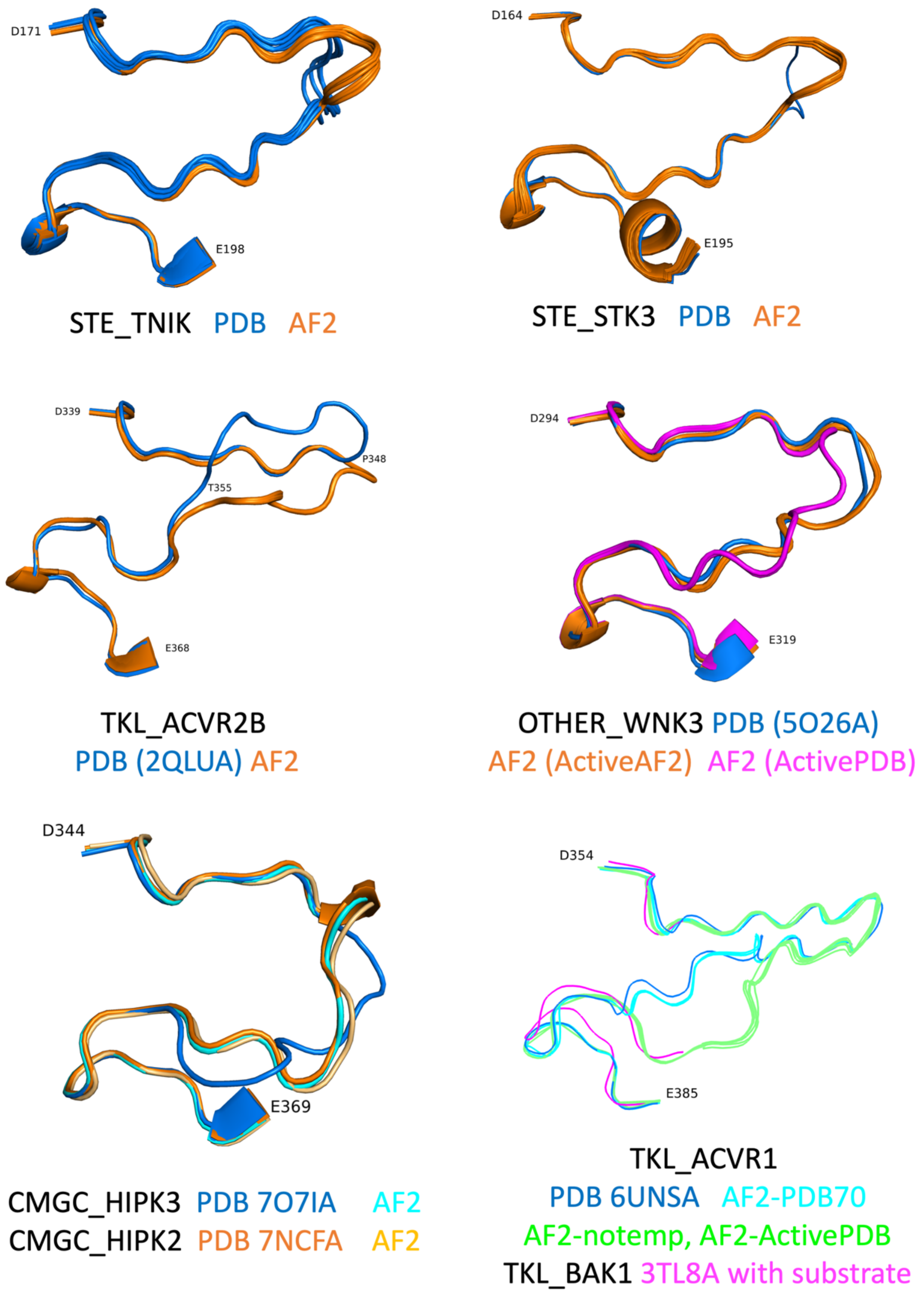

Supplementary Figure 3. Benchmark structures with large RMSD to the best scoring AlphaFold2 models.
STE_TNIK, STE_STK2, and TKL_ACVR2B show deviations between the PDB structures (blue) and AlphaFold2 models (orange) only in the outer regions of the activation loop. For OTHER_WNK3, the highest scoring pLDDT model is from an ActivePDB template (magenta) while an ActiveAF template model is much closer (orange) to the benchmark structure (PDB:5O26A, blue). For CMGC_HIPK3, the AF2 model more closely resembles an active PDB structure of HIPK2 (PDB: 7NCFA, orange) as well as an AF2 model of HIPK2 (light orange) than a PDB structure of HIPK3 itself (7O7IA, blue). The activation loop sequences are identical in 25 of 26 positions, and it is likely that AF2 is correct in modeling very similar conformations of the activation loop. The PDB structure of TKL_ACVR1 (PDB: 6UNSA. blue) resembles the AF2 model made from PDB70 (cyan), while the

bioRxiv preprint doi: https://doi.org/10.1101/2023.07.21.550125; this version posted September 3, 2023. The copyright holder for this preprint (which was not certified by peer review) is the author/funder, who has granted bioRxiv a license to display the preprint in perpetuity. It is made available under aCC-BY 4.0 International license.

ActivePDB AF2 model and the no-template AF2 model more closely resembles a substrate-bound PDB structure of TKL_BAK1 from *Arabidopsis* (PDB: 3TL8, magenta, substrate not shown). It is likely that the ActivePDB AF2 model (green) is a substrate-binding structure, while the PDB structure is not.

bioRxiv preprint doi: https://doi.org/10.1101/2023.07.21.550125; this version posted September 3, 2023. The copyright holder for this preprint (which was not certified by peer review) is the author/funder, who has granted bioRxiv a license to display the preprint in perpetuity. It is made available under aCC-BY 4.0 International license.

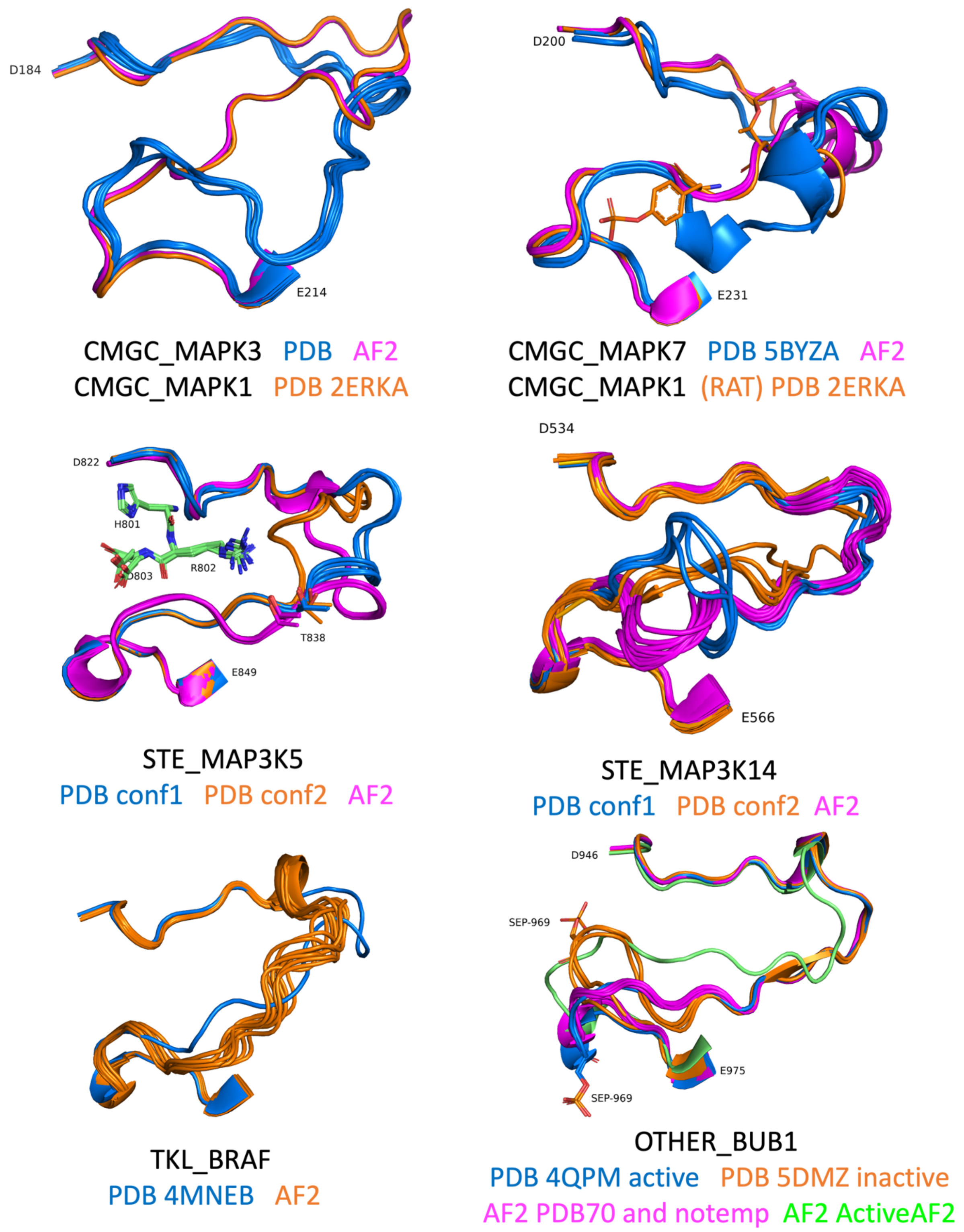

Supplementary Figure 4. Benchmark structures with large RMSD to the best scoring AlphaFold2 models. CMGC_MAPK3 and CMGC_MAPK7 show the same bulge toward the C-terminal end of the activation loop that is present in CMGC_MAPK1 (Figure 13C, main paper) in PDB structures (blue). The AF2 structures (magenta) more closely resemble RAT CMGC_MAPK1 (PDB:2ERKA, orange) shown in both figures. It is likely the AF2 structures are correct substrate-binding forms of MAPK3 and MAPK7. For STE_MAP3K5, the two dominant PDB conformations (conf1 and conf2) are not typical of substrate-binding structures of STE kinases in the PDB. They also differ substantially from each other in the outer portions of the activation loop. The AF2 models (magenta) are closer to substrate-binding structures of STE kinases in Table 1 (PDB:2q0nA, 4zy4A). For STE_MAP3K14, the AF2 structures are quite different than either of the dominant PDB conformations for reasons

bioRxiv preprint doi: https://doi.org/10.1101/2023.07.21.550125; this version posted September 3, 2023. The copyright holder for this preprint (which was not certified by peer review) is the author/funder, who has granted bioRxiv a license to display the preprint in perpetuity. It is made available under aCC-BY 4.0 International license.

that are unknown. TKL_BRAF is not that close to other kinases in the PDB other than RAF1, and the AF2 models are quite different than the benchmark one active structure with a complete activation loop in the PDB (4MNEB). OTHER_BUB1 is also distantly related to all other kinases; in the PDB there are two conformations, one of which has a large bulge blocking the substrate binding site (orange). Most of these structures are not phosphorylated on Ser969, although one of them is (shown in the figure). The other PDB structures (e.g., PDB:4QPM, blue) are more likely to be substrate-capable and most of these structures are phosphorylated on Ser969. The ActiveAF2-template-based model is quite different from both PDB conformations, while the PDB70 and no-template AF2 models (magenta) are much closer to the Active PDB structure (4QPM) and are most likely correct, even though they do not have pLDDT values as high as the ActiveAF2-template structures (green).

bioRxiv preprint doi: https://doi.org/10.1101/2023.07.21.550125; this version posted September 3, 2023. The copyright holder for this preprint (which was not certified by peer review) is the author/funder, who has granted bioRxiv a license to display the preprint in perpetuity. It is made available under aCC-BY 4.0 International license.

Supplementary Table 1. Catalytic kinase domains in the human proteome
The sequence constructs for kinase models produced in this study are given for each human catalytic protein kinase.

N	N (Fam)	Fam_Gene	SwissProt ID	Gene	Uniprot Acc.	Kinase Start	Kinase End	Kinase Length	Length protein
1	1	AGC_AKT1	AKT1_HUMAN	AKT1	P31749	142	416	275	480
2	2	AGC_AKT2	AKT2_HUMAN	AKT2	P31751	144	417	274	481
3	3	AGC_AKT3	AKT3_HUMAN	AKT3	Q9Y243	140	413	274	479
4	4	AGC_CDC42BPA	MRCKA_HUMAN	CDC42BPA	Q5VT25	69	351	283	1732
5	5	AGC_CDC42BPB	MRCKB_HUMAN	CDC42BPB	Q9Y5S2	68	350	283	1711
6	6	AGC_CDC42BPG	MRCKG_HUMAN	CDC42BPG	Q6DT37	63	345	283	1551
7	7	AGC_CIT	CTRO_HUMAN	CIT	O14578	89	368	280	2027
8	8	AGC_DMPK	DMPK_HUMAN	DMPK	Q09013	63	347	285	629
9	9	AGC_GRK1	GRK1_HUMAN	GRK1	Q15835	182	463	282	563
10	10	AGC_GRK2	ARBK1_HUMAN	GRK2	P25098	183	461	279	689
11	11	AGC_GRK3	ARBK2_HUMAN	GRK3	P35626	183	461	279	688
12	12	AGC_GRK4	GRK4_HUMAN	GRK4	P32298	179	457	279	578
13	13	AGC_GRK5	GRK5_HUMAN	GRK5	P34947	178	456	279	590
14	14	AGC_GRK6	GRK6_HUMAN	GRK6	P43250	178	456	279	576
15	15	AGC_GRK7	GRK7_HUMAN	GRK7	Q8WTQ7	183	462	280	553
16	16	AGC_LATS1	LATS1_HUMAN	LATS1	O95835	697	1018	322	1130
17	17	AGC_LATS2	LATS2_HUMAN	LATS2	Q9NRM7	660	981	322	1088
18	18	AGC_MAST1	MAST1_HUMAN	MAST1	Q9Y2H9	366	655	290	1570
19	19	AGC_MAST2	MAST2_HUMAN	MAST2	Q6P0Q8	504	793	290	1798
20	20	AGC_MAST3	MAST3_HUMAN	MAST3	O60307	359	648	290	1309
21	21	AGC_MAST4	MAST4_HUMAN	MAST4	O15021	562	851	290	2623
22	22	AGC_MASTL	GWL_HUMAN	MASTL	Q96GX5	27	843	817	879
23	23	AGC_PDPK1	PDPK1_HUMAN	PDPK1	O15530	74	350	277	556
24	24	AGC_PKN1	PKN1_HUMAN	PKN1	Q16512	607	882	276	942
25	25	AGC_PKN2	PKN2_HUMAN	PKN2	Q16513	649	924	276	984
26	26	AGC_PKN3	PKN3_HUMAN	PKN3	Q6P5Z2	551	826	276	889
27	27	AGC_PRKACA	KAPCA_HUMAN	PRKACA	P17612	36	306	271	351
28	28	AGC_PRKACB	KAPCB_HUMAN	PRKACB	P22694	36	306	271	351
29	29	AGC_PRKACG	KAPCG_HUMAN	PRKACG	P22612	36	306	271	351
30	30	AGC_PRKCA	KPCA_HUMAN	PRKCA	P17252	331	605	275	672
31	31	AGC_PRKCB	KPCB_HUMAN	PRKCB	P05771	334	608	275	671
32	32	AGC_PRKCD	KPCD_HUMAN	PRKCD	Q05655	341	611	271	676
33	33	AGC_PRKCE	KPCE_HUMAN	PRKCE	Q02156	400	676	277	737
34	34	AGC_PRKCG	KPCG_HUMAN	PRKCG	P05129	343	622	280	697
35	35	AGC_PRKCH	KPCL_HUMAN	PRKCH	P24723	347	622	276	683
36	36	AGC_PRKCI	KPCI_HUMAN	PRKCI	P41743	246	530	285	596
37	37	AGC_PRKCQ	KPCT_HUMAN	PRKCQ	Q04759	372	642	271	706
38	38	AGC_PRKCZ	KPCZ_HUMAN	PRKCZ	Q05513	244	526	283	592
39	39	AGC_PRKG1	KGP1_HUMAN	PRKG1	Q13976	352	627	276	671
40	40	AGC_PRKG2	KGP2_HUMAN	PRKG2	Q13237	445	719	275	762
41	41	AGC_PRKX	PRKX_HUMAN	PRKX	P51817	41	311	271	358
42	42	AGC_ROCK1	ROCK1_HUMAN	ROCK1	Q13464	68	346	279	1354
43	43	AGC_ROCK2	ROCK2_HUMAN	ROCK2	O75116	84	362	279	1388
44	44	AGC_RPS6KA1-1	KS6A1_HUMAN	RPS6KA1	Q15418	54	329	276	735
45	45	AGC_RPS6KA2-1	KS6A2_HUMAN	RPS6KA2	Q15349	51	326	276	733
46	46	AGC_RPS6KA3-1	KS6A3_HUMAN	RPS6KA3	P51812	60	335	276	740
47	47	AGC_RPS6KA4-1	KS6A4_HUMAN	RPS6KA4	O75676	25	309	285	772
48	48	AGC_RPS6KA5-1	KS6A5_HUMAN	RPS6KA5	O75582	41	326	286	802
49	49	AGC_RPS6KA6-1	KS6A6_HUMAN	RPS6KA6	Q9UK32	65	338	274	745
50	50	AGC_RPS6KB1	KS6B1_HUMAN	RPS6KB1	P23443	83	360	278	525
51	51	AGC_RPS6KB2	KS6B2_HUMAN	RPS6KB2	Q9UBS0	59	336	278	482
52	52	AGC_RSKR	KS6R_HUMAN	RSKR	Q96LW2	99	367	269	410
53	53	AGC_SGK1	SGK1_HUMAN	SGK1	O00141	90	363	274	431
54	54	AGC_SGK2	SGK2_HUMAN	SGK2	Q9HBY8	87	360	274	367
55	55	AGC_SGK3	SGK3_HUMAN	SGK3	Q96BR1	154	427	274	496
56	56	AGC_STK32A	ST32A_HUMAN	STK32A	Q8WU08	15	289	275	396
57	57	AGC_STK32B	ST32B_HUMAN	STK32B	Q9NY57	15	291	277	414
58	58	AGC_STK32C	ST32C_HUMAN	STK32C	Q86UX6	85	361	277	486
59	59	AGC_STK38	STK38_HUMAN	STK38	Q15208	81	390	310	465
60	60	AGC_STK38L	ST38L_HUMAN	STK38L	Q9Y2H1	82	391	310	464
61	1	CAMK_AURKA	AURKA_HUMAN	AURKA	O14965	125	391	267	403
62	2	CAMK_AURKB	AURKB_HUMAN	AURKB	Q96GD4	69	335	267	344
63	3	CAMK_AURKC	AURKC_HUMAN	AURKC	Q9UQB9	35	301	267	309
64	4	CAMK_BRSK1	BRSK1_HUMAN	BRSK1	Q8TDC3	26	293	268	778
65	5	CAMK_BRSK2	BRSK2_HUMAN	BRSK2	Q8IWQ3	11	278	268	736
66	6	CAMK_CAMK1	KCC1A_HUMAN	CAMK1	Q14012	12	284	273	370
67	7	CAMK_CAMK1D	KCC1D_HUMAN	CAMK1D	Q8IU85	15	287	273	385
68	8	CAMK_CAMK1G	KCC1G_HUMAN	CAMK1G	Q96NX5	15	285	271	476
69	9	CAMK_CAMK2A	KCC2A_HUMAN	CAMK2A	Q9UQM7	5	279	275	478
70	10	CAMK_CAMK2B	KCC2B_HUMAN	CAMK2B	Q13554	6	280	275	666
71	11	CAMK_CAMK2D	KCC2D_HUMAN	CAMK2D	Q13557	6	280	275	499
72	12	CAMK_CAMK2G	KCC2G_HUMAN	CAMK2G	Q13555	6	280	275	558
73	13	CAMK_CAMK4	KCC4_HUMAN	CAMK4	Q16566	38	308	271	473
74	14	CAMK_CAMKK1	KKCC1_HUMAN	CAMKK1	Q8N5S9	120	417	298	505

bioRxiv preprint doi: https://doi.org/10.1101/2023.07.21.550125; this version posted September 3, 2023. The copyright holder for this preprint (which was not certified by peer review) is the author/funder, who has granted bioRxiv a license to display the preprint in perpetuity. It is made available under aCC-BY 4.0 International license.

75	15	CAMK_CAMKK2	KKCC2_HUMAN	CAMKK2	Q96RR4	157	454	298	588
76	16	CAMK_CHEK1	CHK1_HUMAN	CHEK1	O14757	1	273	273	476
77	17	CAMK_CHEK2	CHK2_HUMAN	CHEK2	O96017	212	494	283	543
78	18	CAMK_DAPK1	DAPK1_HUMAN	DAPK1	P53355	5	283	279	1430
79	19	CAMK_DAPK2	DAPK2_HUMAN	DAPK2	Q9UIK4	15	293	279	370
80	20	CAMK_DAPK3	DAPK3_HUMAN	DAPK3	O43293	5	283	279	454
81	21	CAMK_DCLK1	DCLK1_HUMAN	DCLK1	O15075	382	655	274	740
82	22	CAMK_DCLK2	DCLK2_HUMAN	DCLK2	Q8N568	386	659	274	766
83	23	CAMK_DCLK3	DCLK3_HUMAN	DCLK3	Q9C098	348	621	274	648
84	24	CAMK_HUNK	HUNK_HUMAN	HUNK	P57058	54	328	275	714
85	25	CAMK_KALRN	KALRN_HUMAN	KALRN	O60229	2675	2945	271	2986
86	26	CAMK_MAPKAPK2	MAPK2_HUMAN	MAPKAPK2	P49137	55	333	279	400
87	27	CAMK_MAPKAPK3	MAPK3_HUMAN	MAPKAPK3	Q16644	35	312	278	382
88	28	CAMK_MAPKAPK5	MAPK5_HUMAN	MAPKAPK5	Q8IW41	12	312	301	473
89	29	CAMK_MARK1	MARK1_HUMAN	MARK1	Q9P0L2	52	319	268	795
90	30	CAMK_MARK2	MARK2_HUMAN	MARK2	Q7KZI7	45	312	268	788
91	31	CAMK_MARK3	MARK3_HUMAN	MARK3	P27448	48	315	268	753
92	32	CAMK_MARK4	MARK4_HUMAN	MARK4	Q96L34	51	318	268	752
93	33	CAMK_MELK	MELK_HUMAN	MELK	Q14680	3	271	269	651
94	34	CAMK_MKNK1	MKNK1_HUMAN	MKNK1	Q9BUB5	40	382	343	465
95	35	CAMK_MKNK2	MKNK2_HUMAN	MKNK2	Q9HBH9	75	376	302	465
96	36	CAMK_MYLK	MYLK_HUMAN	MYLK	Q15746	1456	1727	272	1914
97	37	CAMK_MYLK2	MYLK2_HUMAN	MYLK2	Q9H1R3	275	548	274	596
98	38	CAMK_MYLK3	MYLK3_HUMAN	MYLK3	Q32MK0	505	778	274	819
99	39	CAMK_MYLK4	MYLK4_HUMAN	MYLK4	Q86YV6	96	369	274	388
100	40	CAMK_NIM1K	NIM1_HUMAN	NIM1K	Q8IY84	66	333	268	436
101	41	CAMK_NUAK1	NUAK1_HUMAN	NUAK1	O60285	47	314	268	661
102	42	CAMK_NUAK2	NUAK2_HUMAN	NUAK2	Q9H093	45	311	267	628
103	43	CAMK_OBSCN−1	OBSCN_HUMAN	OBSCN	Q5VST9	6460	6729	270	7968
104	44	CAMK_OBSCN−2	OBSCN_HUMAN	OBSCN	Q5VST9	7664	7932	269	7968
105	45	CAMK_PASK	PASK_HUMAN	PASK	Q96RG2	991	1259	269	1323
106	46	CAMK_PHKG1	PHKG1_HUMAN	PHKG1	Q16816	12	296	285	387
107	47	CAMK_PHKG2	PHKG2_HUMAN	PHKG2	P15735	16	299	284	406
108	48	CAMK_PIM1	PIM1_HUMAN	PIM1	P11309	30	298	269	313
109	49	CAMK_PIM2	PIM2_HUMAN	PIM2	Q9P1W9	24	294	271	311
110	50	CAMK_PIM3	PIM3_HUMAN	PIM3	Q86V86	32	301	270	326
111	51	CAMK_PLK1	PLK1_HUMAN	PLK1	P53350	45	313	269	603
112	52	CAMK_PLK2	PLK2_HUMAN	PLK2	Q9NYY3	74	342	269	685
113	53	CAMK_PLK3	PLK3_HUMAN	PLK3	Q9H4B4	54	322	269	646
114	54	CAMK_PLK4	PLK4_HUMAN	PLK4	O00444	4	273	270	970
115	55	CAMK_PNCK	KCC1B_HUMAN	PNCK	Q6P2M8	7	278	272	343
116	56	CAMK_PRKAA1	AAPK1_HUMAN	PRKAA1	Q13131	19	287	269	559
117	57	CAMK_PRKAA2	AAPK2_HUMAN	PRKAA2	P54646	8	276	269	552
118	58	CAMK_PRKD1	KPCD1_HUMAN	PRKD1	Q15139	575	847	273	912
119	59	CAMK_PRKD2	KPCD2_HUMAN	PRKD2	Q9BZL6	543	815	273	878
120	60	CAMK_PRKD3	KPCD3_HUMAN	PRKD3	O94806	568	840	273	890
121	61	CAMK_PSKH1	KPSH1_HUMAN	PSKH1	P11801	90	363	274	424
122	62	CAMK_RPS6KA1−2	KS6A1_HUMAN	RPS6KA1	Q15418	410	683	274	735
123	63	CAMK_RPS6KA2−2	KS6A2_HUMAN	RPS6KA2	Q15349	407	680	274	733
124	64	CAMK_RPS6KA3−2	KS6A3_HUMAN	RPS6KA3	P51812	414	687	274	740
125	65	CAMK_RPS6KA4−2	KS6A4_HUMAN	RPS6KA4	O75676	400	682	283	772
126	66	CAMK_RPS6KA5−2	KS6A5_HUMAN	RPS6KA5	O75582	415	695	281	802
127	67	CAMK_RPS6KA6−2	KS6A6_HUMAN	RPS6KA6	Q9UK32	418	691	274	745
128	68	CAMK_SIK1	SIK1_HUMAN	SIK1	P57059	19	286	268	783
129	69	CAMK_SIK2	SIK2_HUMAN	SIK2	Q9H0K1	12	279	268	926
130	70	CAMK_SIK3	SIK3_HUMAN	SIK3	Q9Y2K2	58	325	268	1321
131	71	CAMK_SNRK	SNRK_HUMAN	SNRK	Q9NRH2	8	277	270	765
132	72	CAMK_SPEG−1	SPEG_HUMAN	SPEG	Q15772	1593	1862	270	3267
133	73	CAMK_SPEG−2	SPEG_HUMAN	SPEG	Q15772	2958	3226	269	3267
134	74	CAMK_STK11	STK11_HUMAN	STK11	Q15831	41	317	277	433
135	75	CAMK_STK17A	ST17A_HUMAN	STK17A	Q9UEE5	51	329	279	414
136	76	CAMK_STK17B	ST17B_HUMAN	STK17B	O94768	24	301	278	372
137	77	CAMK_STK33	STK33_HUMAN	STK33	Q9BYT3	108	389	282	514
138	78	CAMK_TRIO	TRIO_HUMAN	TRIO	O75962	2788	3058	271	3097
139	79	CAMK_TSSK1B	TSSK1_HUMAN	TSSK1B	Q9BXA7	4	280	277	367
140	80	CAMK_TSSK2	TSSK2_HUMAN	TSSK2	Q96PF2	4	280	277	358
141	81	CAMK_TSSK3	TSSK3_HUMAN	TSSK3	Q96PN8	2	268	267	268
142	82	CAMK_TSSK4	TSSK4_HUMAN	TSSK4	Q6SA08	17	301	285	328
143	83	CAMK_TSSK6	TSSK6_HUMAN	TSSK6	Q9BXA6	4	273	270	273
144	1	CK1_CSNK1A1	KC1A_HUMAN	CSNK1A1	P48729	9	291	283	337
145	2	CK1_CSNK1A1L	KC1AL_HUMAN	CSNK1A1L	Q8N752	9	291	283	337
146	3	CK1_CSNK1D	KC1D_HUMAN	CSNK1D	P48730	1	283	283	415
147	4	CK1_CSNK1E	KC1E_HUMAN	CSNK1E	P49674	1	283	283	416
148	5	CK1_CSNK1G1	KC1G1_HUMAN	CSNK1G1	Q9HCP0	36	321	286	422
149	6	CK1_CSNK1G2	KC1G2_HUMAN	CSNK1G2	P78368	38	322	285	415
150	7	CK1_CSNK1G3	KC1G3_HUMAN	CSNK1G3	Q9Y6M4	35	318	284	447
151	8	CK1_TTBK1	TTBK1_HUMAN	TTBK1	Q5TCY1	26	303	278	1321
152	9	CK1_TTBK2	TTBK2_HUMAN	TTBK2	Q6IQ55	13	290	278	1244
153	10	CK1_VRK1	VRK1_HUMAN	VRK1	Q99986	29	334	306	396
154	11	CK1_VRK2	VRK2_HUMAN	VRK2	Q86Y07	21	323	303	508
155	1	CMGC_CDK1	CDK1_HUMAN	CDK1	P06493	1	295	295	297
156	2	CMGC_CDK10	CDK10_HUMAN	CDK10	Q15131	31	331	301	360
157	3	CMGC_CDK11A	CD11A_HUMAN	CDK11A	Q9UQ88	418	719	302	783

bioRxiv preprint doi: https://doi.org/10.1101/2023.07.21.550125; this version posted September 3, 2023. The copyright holder for this preprint (which was not certified by peer review) is the author/funder, who has granted bioRxiv a license to display the preprint in perpetuity. It is made available under aCC-BY 4.0 International license.

158	4	CMGC_CDK11B	CD11B_HUMAN	CDK11B	P21127	430	731	302	795
159	5	CMGC_CDK12	CDK12_HUMAN	CDK12	Q9NYV4	719	1028	310	1490
160	6	CMGC_CDK13	CDK13_HUMAN	CDK13	Q14004	697	1006	310	1512
161	7	CMGC_CDK14	CDK14_HUMAN	CDK14	O94921	127	427	301	469
162	8	CMGC_CDK15	CDK15_HUMAN	CDK15	Q96Q40	95	395	301	435
163	9	CMGC_CDK16	CDK16_HUMAN	CDK16	Q00536	157	454	298	496
164	10	CMGC_CDK17	CDK17_HUMAN	CDK17	Q00537	184	481	298	523
165	11	CMGC_CDK18	CDK18_HUMAN	CDK18	Q07002	136	433	298	474
166	12	CMGC_CDK19	CDK19_HUMAN	CDK19	Q9BWU1	12	343	332	502
167	13	CMGC_CDK2	CDK2_HUMAN	CDK2	P24941	1	294	294	298
168	14	CMGC_CDK20	CDK20_HUMAN	CDK20	Q8IZL9	1	296	296	346
169	15	CMGC_CDK3	CDK3_HUMAN	CDK3	Q00526	1	294	294	305
170	16	CMGC_CDK4	CDK4_HUMAN	CDK4	P11802	1	303	303	303
171	17	CMGC_CDK5	CDK5_HUMAN	CDK5	Q00535	1	292	292	292
172	18	CMGC_CDK6	CDK6_HUMAN	CDK6	Q00534	5	308	304	326
173	19	CMGC_CDK7	CDK7_HUMAN	CDK7	P50613	4	304	301	346
174	20	CMGC_CDK8	CDK8_HUMAN	CDK8	P49336	12	343	332	464
175	21	CMGC_CDK9	CDK9_HUMAN	CDK9	P50750	11	323	313	372
176	22	CMGC_CDKL1	CDKL1_HUMAN	CDKL1	Q00532	1	296	296	358
177	23	CMGC_CDKL2	CDKL2_HUMAN	CDKL2	Q92772	1	295	295	493
178	24	CMGC_CDKL3	CDKL3_HUMAN	CDKL3	Q8IVW4	1	294	294	592
179	25	CMGC_CDKL4	CDKL4_HUMAN	CDKL4	Q5MAI5	1	294	294	379
180	26	CMGC_CDKL5	CDKL5_HUMAN	CDKL5	O76039	5	305	301	960
181	27	CMGC_CLK1	CLK1_HUMAN	CLK1	P49759	153	484	332	484
182	28	CMGC_CLK2	CLK2_HUMAN	CLK2	P49760	155	487	333	499
183	29	CMGC_CLK3	CLK3_HUMAN	CLK3	P49761	296	628	333	638
184	30	CMGC_CLK4	CLK4_HUMAN	CLK4	Q9HAZ1	151	481	331	481
185	31	CMGC_CSNK2A1	CSK21_HUMAN	CSNK2A1	P68400	31	332	302	391
186	32	CMGC_CSNK2A2	CSK22_HUMAN	CSNK2A2	P19784	32	333	302	350
187	33	CMGC_CSNK2A3	CSK23_HUMAN	CSNK2A3	Q8NEV1	31	332	302	391
188	34	CMGC_DYRK1A	DYR1A_HUMAN	DYRK1A	Q13627	151	487	337	763
189	35	CMGC_DYRK1B	DYR1B_HUMAN	DYRK1B	Q9Y463	103	439	337	629
190	36	CMGC_DYRK2	DYRK2_HUMAN	DYRK2	Q92630	214	543	330	601
191	37	CMGC_DYRK3	DYRK3_HUMAN	DYRK3	O43781	201	530	330	588
192	38	CMGC_DYRK4	DYRK4_HUMAN	DYRK4	Q9NR20	96	408	313	520
193	39	CMGC_GSK3A	GSK3A_HUMAN	GSK3A	P49840	111	411	301	483
194	40	CMGC_GSK3B	GSK3B_HUMAN	GSK3B	P49841	48	348	301	420
195	41	CMGC_HIPK1	HIPK1_HUMAN	HIPK1	Q86Z02	182	526	345	1210
196	42	CMGC_HIPK2	HIPK2_HUMAN	HIPK2	Q9H2X6	191	535	345	1198
197	43	CMGC_HIPK3	HIPK3_HUMAN	HIPK3	Q9H422	189	533	345	1215
198	44	CMGC_HIPK4	HIPK4_HUMAN	HIPK4	Q8NE63	3	355	353	616
199	45	CMGC_ICK	CILK1_HUMAN	CILK1	Q9UPZ9	1	292	292	632
200	46	CMGC_MAK	MAK_HUMAN	MAK	P20794	1	292	292	623
201	47	CMGC_MAPK1	MK01_HUMAN	MAPK1	P28482	17	321	305	360
202	48	CMGC_MAPK10	MK10_HUMAN	MAPK10	P53779	56	367	312	464
203	49	CMGC_MAPK11	MK11_HUMAN	MAPK11	Q15759	16	316	301	364
204	50	CMGC_MAPK12	MK12_HUMAN	MAPK12	P53778	19	319	301	367
205	51	CMGC_MAPK13	MK13_HUMAN	MAPK13	O15264	17	316	300	365
206	52	CMGC_MAPK14	MK14_HUMAN	MAPK14	Q16539	16	316	301	360
207	53	CMGC_MAPK15	MK15_HUMAN	MAPK15	Q8TD08	5	312	308	544
208	54	CMGC_MAPK3	MK03_HUMAN	MAPK3	P27361	34	338	305	379
209	55	CMGC_MAPK4	MK04_HUMAN	MAPK4	P31152	12	320	309	587
210	56	CMGC_MAPK6	MK06_HUMAN	MAPK6	Q16659	12	324	313	721
211	57	CMGC_MAPK7	MK07_HUMAN	MAPK7	Q13164	47	355	309	816
212	58	CMGC_MAPK8	MK08_HUMAN	MAPK8	P45983	18	329	312	427
213	59	CMGC_MAPK9	MK09_HUMAN	MAPK9	P45984	18	329	312	424
214	60	CMGC_MOK	MOK_HUMAN	MOK	Q9UQ07	1	293	293	419
215	61	CMGC_NLK	NLK_HUMAN	NLK	Q9UBE8	130	435	306	527
216	62	CMGC_PRPF4B	PRP4B_HUMAN	PRPF4B	Q13523	679	1007	329	1007
217	63	CMGC_SRPK1	SRPK1_HUMAN	SRPK1	Q96SB4	72	655	584	655
218	64	CMGC_SRPK2	SRPK2_HUMAN	SRPK2	P78362	73	688	616	688
219	65	CMGC_SRPK3	SRPK3_HUMAN	SRPK3	Q9UPE1	71	567	497	567
220	1	NEK_NEK1	NEK1_HUMAN	NEK1	Q96PY6	1	266	266	1258
221	2	NEK_NEK10	NEK10_HUMAN	NEK10	Q6ZWH5	511	793	283	1172
222	3	NEK_NEK11	NEK11_HUMAN	NEK11	Q8NG66	21	295	275	645
223	4	NEK_NEK2	NEK2_HUMAN	NEK2	P51955	1	279	279	445
224	5	NEK_NEK3	NEK3_HUMAN	NEK3	P51956	1	265	265	506
225	6	NEK_NEK4	NEK4_HUMAN	NEK4	P51957	1	269	269	841
226	7	NEK_NEK5	NEK5_HUMAN	NEK5	Q6P3R8	1	267	267	708
227	8	NEK_NEK6	NEK6_HUMAN	NEK6	Q9HC98	37	313	277	313
228	9	NEK_NEK7	NEK7_HUMAN	NEK7	Q8TDX7	26	302	277	302
229	10	NEK_NEK8	NEK8_HUMAN	NEK8	Q86SG6	1	266	266	692
230	11	NEK_NEK9	NEK9_HUMAN	NEK9	Q8TD19	44	316	273	979
231	1	OTHER_AAK1	AAK1_HUMAN	AAK1	Q2M2I8	38	321	284	961
232	2	OTHER_BMP2K	BMP2K_HUMAN	BMP2K	Q9NSY1	43	325	283	1161
233	3	OTHER_BUB1	BUB1_HUMAN	BUB1	O43683	779	1064	286	1085
234	4	OTHER_CDC7	CDC7_HUMAN	CDC7	O00311	50	574	525	574
235	5	OTHER_CHUK	IKKA_HUMAN	CHUK	O15111	7	317	311	745
236	6	OTHER_DSTYK	DUSTY_HUMAN	DSTYK	Q6XUX3	640	916	277	929
237	7	OTHER_EIF2AK1	E2AK1_HUMAN	EIF2AK1	Q9BQI3	159	591	433	630
238	8	OTHER_EIF2AK2	E2AK2_HUMAN	EIF2AK2	P19525	259	546	288	551
239	9	OTHER_EIF2AK3	E2AK3_HUMAN	EIF2AK3	Q9NZJ5	585	1085	501	1116
240	10	OTHER_EIF2AK4-2	E2AK4_HUMAN	EIF2AK4	Q9P2K8	582	1009	428	1649

bioRxiv preprint doi: https://doi.org/10.1101/2023.07.21.550125; this version posted September 3, 2023. The copyright holder for this preprint (which was not certified by peer review) is the author/funder, who has granted bioRxiv a license to display the preprint in perpetuity. It is made available under aCC-BY 4.0 International license.

241	11	OTHER_ERN1	ERN1_HUMAN	ERN1	O75460	561	840	280	977
242	12	OTHER_ERN2	ERN2_HUMAN	ERN2	Q76MJ5	510	789	280	926
243	13	OTHER_GAK	GAK_HUMAN	GAK	O14976	32	323	292	1311
244	14	OTHER_HASPIN	HASP_HUMAN	GSG2	Q8TF76	476	798	323	798
245	15	OTHER_IKBKB	IKKB_HUMAN	IKBKB	O14920	7	316	310	756
246	16	OTHER_IKBKE	IKKE_HUMAN	IKBKE	Q14164	1	312	312	716
247	17	OTHER_MOS	MOS_HUMAN	MOS	P00540	52	346	295	346
248	18	OTHER_PBK	TOPK_HUMAN	PBK	Q96KB5	24	322	299	322
249	19	OTHER_PDIK1L	PDK1L_HUMAN	PDIK1L	Q8N165	1	339	339	341
250	20	OTHER_PINK1	PINK1_HUMAN	PINK1	Q9BXM7	148	517	370	581
251	21	OTHER_PKDCC	PKDCC_HUMAN	PKDCC	Q504Y2	130	399	270	493
252	22	OTHER_PKMYT1	PMYT1_HUMAN	PKMYT1	Q99640	102	367	266	499
253	23	OTHER_SBK1	SBK1_HUMAN	SBK1	Q52WX2	45	323	279	424
254	24	OTHER_SBK2	SBK2_HUMAN	SBK2	P0C263	54	335	282	348
255	25	OTHER_SBK3	SBK3_HUMAN	SBK3	P0C264	35	314	280	359
256	26	OTHER_STK16	STK16_HUMAN	STK16	O75716	12	300	289	305
257	27	OTHER_STK35	STK35_HUMAN	STK35	Q8TDR2	194	534	341	534
258	28	OTHER_STK36	STK36_HUMAN	STK36	Q9NRP7	1	262	262	1315
259	29	OTHER_TBK1	TBK1_HUMAN	TBK1	Q9UHD2	1	312	312	729
260	30	OTHER_TLK1	TLK1_HUMAN	TLK1	Q9UKI8	448	742	295	766
261	31	OTHER_TLK2	TLK2_HUMAN	TLK2	Q86UE8	454	749	296	772
262	32	OTHER_TP53RK	PRPK_HUMAN	TP53RK	Q96S44	25	253	229	253
263	33	OTHER_TTK	TTK_HUMAN	TTK	P33981	517	799	283	857
264	34	OTHER_UHMK1	UHMK1_HUMAN	UHMK1	Q8TAS1	15	312	298	419
265	35	OTHER_ULK1	ULK1_HUMAN	ULK1	O75385	6	286	281	1050
266	36	OTHER_ULK2	ULK2_HUMAN	ULK2	Q8IYT8	1	279	279	1036
267	37	OTHER_ULK3	ULK3_HUMAN	ULK3	Q6PHR2	6	278	273	472
268	38	OTHER_WEE1	WEE1_HUMAN	WEE1	P30291	291	577	287	646
269	39	OTHER_WEE2	WEE2_HUMAN	WEE2	P0C1S8	204	494	291	567
270	40	OTHER_WNK1	WNK1_HUMAN	WNK1	Q9H4A3	212	487	276	2382
271	41	OTHER_WNK2	WNK2_HUMAN	WNK2	Q9Y3S1	186	461	276	2297
272	42	OTHER_WNK3	WNK3_HUMAN	WNK3	Q9BYP7	138	413	276	1800
273	43	OTHER_WNK4	WNK4_HUMAN	WNK4	Q96J92	165	440	276	1243
274	1	STE_MAP2K1	MP2K1_HUMAN	MAP2K1	Q02750	60	369	310	393
275	2	STE_MAP2K2	MP2K2_HUMAN	MAP2K2	P36507	64	377	314	400
276	3	STE_MAP2K3	MP2K3_HUMAN	MAP2K3	P46734	56	333	278	347
277	4	STE_MAP2K4	MP2K4_HUMAN	MAP2K4	P45985	94	375	282	399
278	5	STE_MAP2K5	MP2K5_HUMAN	MAP2K5	Q13163	158	427	270	448
279	6	STE_MAP2K6	MP2K6_HUMAN	MAP2K6	P52564	45	322	278	334
280	7	STE_MAP2K7	MP2K7_HUMAN	MAP2K7	O14733	112	388	277	419
281	8	STE_MAP3K1	M3K1_HUMAN	MAP3K1	Q13233	1235	1512	278	1512
282	9	STE_MAP3K14	M3K14_HUMAN	MAP3K14	Q99558	391	661	271	947
283	10	STE_MAP3K15	M3K15_HUMAN	MAP3K15	Q6ZN16	637	916	280	1313
284	11	STE_MAP3K19	M3K19_HUMAN	MAP3K19	Q56UN5	1053	1328	276	1328
285	12	STE_MAP3K2	M3K2_HUMAN	MAP3K2	Q9Y2U5	348	619	272	619
286	13	STE_MAP3K3	M3K3_HUMAN	MAP3K3	Q99759	354	626	273	626
287	14	STE_MAP3K4	M3K4_HUMAN	MAP3K4	Q9Y6R4	1335	1608	274	1608
288	15	STE_MAP3K5	M3K5_HUMAN	MAP3K5	Q99683	665	946	282	1374
289	16	STE_MAP3K6	M3K6_HUMAN	MAP3K6	O95382	635	914	280	1288
290	17	STE_MAP3K8	M3K8_HUMAN	MAP3K8	P41279	119	396	278	467
291	18	STE_MAP4K1	M4K1_HUMAN	MAP4K1	Q92918	9	282	274	833
292	19	STE_MAP4K2	M4K2_HUMAN	MAP4K2	Q12851	8	281	274	820
293	20	STE_MAP4K3	M4K3_HUMAN	MAP4K3	Q8IVH8	8	281	274	894
294	21	STE_MAP4K4	M4K4_HUMAN	MAP4K4	O95819	17	297	281	1239
295	22	STE_MAP4K5	M4K5_HUMAN	MAP4K5	Q9Y4K4	12	285	274	846
296	23	STE_MINK1	MINK1_HUMAN	MINK1	Q8N4C8	17	297	281	1332
297	24	STE_MYO3A	MYO3A_HUMAN	MYO3A	Q8NEV4	13	295	283	1616
298	25	STE_MYO3B	MYO3B_HUMAN	MYO3B	Q8WXR4	19	301	283	1341
299	26	STE_NRK	NRK_HUMAN	NRK	Q7Z2Y5	17	321	305	1582
300	27	STE_OXSR1	OXSR1_HUMAN	OXSR1	O95747	9	299	291	527
301	28	STE_PAK1	PAK1_HUMAN	PAK1	Q13153	262	529	268	545
302	29	STE_PAK2	PAK2_HUMAN	PAK2	Q13177	241	507	267	524
303	30	STE_PAK3	PAK3_HUMAN	PAK3	O75914	275	542	268	559
304	31	STE_PAK4	PAK4_HUMAN	PAK4	O96013	313	580	268	591
305	32	STE_PAK5	PAK5_HUMAN	PAK5	Q9P286	441	708	268	719
306	33	STE_PAK6	PAK6_HUMAN	PAK6	Q9NQU5	399	666	268	681
307	34	STE_SLK	SLK_HUMAN	SLK	Q9H2G2	26	300	275	1235
308	35	STE_STK10	STK10_HUMAN	STK10	O94804	28	302	275	968
309	36	STE_STK24	STK24_HUMAN	STK24	Q9Y6E0	28	294	267	443
310	37	STE_STK25	STK25_HUMAN	STK25	O00506	12	278	267	426
311	38	STE_STK26	STK26_HUMAN	STK26	Q9P289	16	282	267	416
312	39	STE_STK3	STK3_HUMAN	STK3	Q13188	19	286	268	491
313	40	STE_STK39	STK39_HUMAN	STK39	Q9UEW8	55	345	291	545
314	41	STE_STK4	STK4_HUMAN	STK4	Q13043	22	289	268	487
315	42	STE_TAOK1	TAOK1_HUMAN	TAOK1	Q7L7X3	20	289	270	1001
316	43	STE_TAOK2	TAOK2_HUMAN	TAOK2	Q9UL54	20	289	270	1235
317	44	STE_TAOK3	TAOK3_HUMAN	TAOK3	Q9H2K8	16	285	270	898
318	45	STE_TNIK	TNIK_HUMAN	TNIK	Q9UKE5	17	297	281	1360
319	1	TKL_ACVR1	ACVR1_HUMAN	ACVR1	Q04771	200	505	306	509
320	2	TKL_ACVR1B	ACV1B_HUMAN	ACVR1B	P36896	199	504	306	505
321	3	TKL_ACVR1C	ACV1C_HUMAN	ACVR1C	Q8NER5	187	492	306	493
322	4	TKL_ACVR2A	AVR2A_HUMAN	ACVR2A	P27037	184	489	306	513
323	5	TKL_ACVR2B	AVR2B_HUMAN	ACVR2B	Q13705	182	488	307	512

bioRxiv preprint doi: https://doi.org/10.1101/2023.07.21.550125; this version posted September 3, 2023. The copyright holder for this preprint (which was not certified by peer review) is the author/funder, who has granted bioRxiv a license to display the preprint in perpetuity. It is made available under aCC-BY 4.0 International license.

324	6	TKL_ACVRL1	ACVL1_HUMAN	ACVRL1	P37023	194	499	306	503
325	7	TKL_AMHR2	AMHR2_HUMAN	AMHR2	Q16671	195	515	321	573
326	8	TKL_ANKK1	ANKK1_HUMAN	ANKK1	Q8NFD2	13	295	283	765
327	9	TKL_ARAF	ARAF_HUMAN	ARAF	P10398	302	577	276	606
328	10	TKL_BMPR1A	BMR1A_HUMAN	BMPR1A	P36894	226	531	306	532
329	11	TKL_BMPR1B	BMR1B_HUMAN	BMPR1B	O00238	196	501	306	502
330	12	TKL_BMPR2	BMPR2_HUMAN	BMPR2	Q13873	195	511	317	1038
331	13	TKL_BRAF	BRAF_HUMAN	BRAF	P15056	449	724	276	766
332	14	TKL_IRAK1	IRAK1_HUMAN	IRAK1	P51617	199	529	331	712
333	15	TKL_IRAK4	IRAK4_HUMAN	IRAK4	Q9NWZ3	167	460	294	460
334	16	TKL_LIMK1	LIMK1_HUMAN	LIMK1	P53667	331	614	284	647
335	17	TKL_LIMK2	LIMK2_HUMAN	LIMK2	P53671	323	611	289	638
336	18	TKL_LRRK1	LRRK1_HUMAN	LRRK1	Q38SD2	1230	1530	301	2015
337	19	TKL_LRRK2	LRRK2_HUMAN	LRRK2	Q5S007	1867	2142	276	2527
338	20	TKL_MAP3K10	M3K10_HUMAN	MAP3K10	Q02779	90	367	278	954
339	21	TKL_MAP3K11	M3K11_HUMAN	MAP3K11	Q16584	109	386	278	847
340	22	TKL_MAP3K12	M3K12_HUMAN	MAP3K12	Q12852	117	374	258	859
341	23	TKL_MAP3K13	M3K13_HUMAN	MAP3K13	O43283	160	417	258	966
342	24	TKL_MAP3K20	M3K20_HUMAN	MAP3K20	Q9NYL2	8	270	263	800
343	25	TKL_MAP3K21	M3K21_HUMAN	MAP3K21	Q5TCX8	116	408	293	1036
344	26	TKL_MAP3K7	M3K7_HUMAN	MAP3K7	O43318	28	294	267	606
345	27	TKL_MAP3K9	M3K9_HUMAN	MAP3K9	P80192	136	413	278	1104
346	28	TKL_RAF1	RAF1_HUMAN	RAF1	P04049	341	616	276	648
347	29	TKL_RIPK1	RIPK1_HUMAN	RIPK1	Q13546	9	295	287	671
348	30	TKL_RIPK2	RIPK2_HUMAN	RIPK2	O43353	10	300	291	540
349	31	TKL_RIPK3	RIPK3_HUMAN	RIPK3	Q9Y572	13	293	281	518
350	32	TKL_RIPK4	RIPK4_HUMAN	RIPK4	P57078	14	293	280	832
351	33	TKL_TESK1	TESK1_HUMAN	TESK1	Q15569	46	321	276	626
352	34	TKL_TESK2	TESK2_HUMAN	TESK2	Q96S53	48	319	272	571
353	35	TKL_TGFBR1	TGFR1_HUMAN	TGFBR1	P36897	197	502	306	503
354	36	TKL_TGFBR2	TGFR2_HUMAN	TGFBR2	P37173	236	548	313	567
355	37	TKL_TNNI3K	TNI3K_HUMAN	TNNI3K	Q59H18	455	729	275	835
356	1	TYR_AATK	LMTK1_HUMAN	AATK	Q6ZMQ8	117	405	289	1374
357	2	TYR_ABL1	ABL1_HUMAN	ABL1	P00519	234	503	270	1130
358	3	TYR_ABL2	ABL2_HUMAN	ABL2	P42684	280	549	270	1182
359	4	TYR_ALK	ALK_HUMAN	ALK	Q9UM73	1108	1393	286	1620
360	5	TYR_AXL	UFO_HUMAN	AXL	P30530	528	813	286	894
361	6	TYR_BLK	BLK_HUMAN	BLK	P51451	233	500	268	505
362	7	TYR_BMX	BMX_HUMAN	BMX	P51813	409	675	267	675
363	8	TYR_BTK	BTK_HUMAN	BTK	Q06187	394	659	266	659
364	9	TYR_CSF1R	CSF1R_HUMAN	CSF1R	P07333	574	920	347	972
365	10	TYR_CSK	CSK_HUMAN	CSK	P41240	187	450	264	450
366	11	TYR_DDR1	DDR1_HUMAN	DDR1	Q08345	602	913	312	913
367	12	TYR_DDR2	DDR2_HUMAN	DDR2	Q16832	555	855	301	855
368	13	TYR_EGFR	EGFR_HUMAN	EGFR	P00533	704	978	275	1210
369	14	TYR_EPHA1	EPHA1_HUMAN	EPHA1	P21709	616	890	275	976
370	15	TYR_EPHA2	EPHA2_HUMAN	EPHA2	P29317	605	881	277	976
371	16	TYR_EPHA3	EPHA3_HUMAN	EPHA3	P29320	613	888	276	983
372	17	TYR_EPHA4	EPHA4_HUMAN	EPHA4	P54764	613	888	276	986
373	18	TYR_EPHA5	EPHA5_HUMAN	EPHA5	P54756	667	942	276	1037
374	19	TYR_EPHA6	EPHA6_HUMAN	EPHA6	Q9UF33	623	940	318	1036
375	20	TYR_EPHA7	EPHA7_HUMAN	EPHA7	Q15375	625	900	276	998
376	21	TYR_EPHA8	EPHA8_HUMAN	EPHA8	P29322	627	902	276	1005
377	22	TYR_EPHB1	EPHB1_HUMAN	EPHB1	P54762	611	888	278	984
378	23	TYR_EPHB2	EPHB2_HUMAN	EPHB2	P29323	613	890	278	1055
379	24	TYR_EPHB3	EPHB3_HUMAN	EPHB3	P54753	625	902	278	998
380	25	TYR_EPHB4	EPHB4_HUMAN	EPHB4	P54760	607	884	278	987
381	26	TYR_ERBB2	ERBB2_HUMAN	ERBB2	P04626	712	986	275	1255
382	27	TYR_ERBB4	ERBB4_HUMAN	ERBB4	Q15303	710	984	275	1308
383	28	TYR_FER	FER_HUMAN	FER	P16591	555	822	268	822
384	29	TYR_FES	FES_HUMAN	FES	P07332	553	822	270	822
385	30	TYR_FGFR1	FGFR1_HUMAN	FGFR1	P11362	470	764	295	822
386	31	TYR_FGFR2	FGFR2_HUMAN	FGFR2	P21802	473	767	295	821
387	32	TYR_FGFR3	FGFR3_HUMAN	FGFR3	P22607	464	758	295	806
388	33	TYR_FGFR4	FGFR4_HUMAN	FGFR4	P22455	459	753	295	802
389	34	TYR_FGR	FGR_HUMAN	FGR	P09769	255	522	268	529
390	35	TYR_FLT1	VGFR1_HUMAN	FLT1	P17948	819	1164	346	1338
391	36	TYR_FLT3	FLT3_HUMAN	FLT3	P36888	602	953	352	993
392	37	TYR_FLT4	VGFR3_HUMAN	FLT4	P35916	837	1179	343	1363
393	38	TYR_FRK	FRK_HUMAN	FRK	P42685	226	497	272	505
394	39	TYR_FYN	FYN_HUMAN	FYN	P06241	263	530	268	537
395	40	TYR_HCK	HCK_HUMAN	HCK	P08631	254	521	268	526
396	41	TYR_IGF1R	IGF1R_HUMAN	IGF1R	P08069	991	1276	286	1367
397	42	TYR_INSR	INSR_HUMAN	INSR	P06213	1015	1300	286	1382
398	43	TYR_INSRR	INSRR_HUMAN	INSRR	P14616	971	1256	286	1297
399	44	TYR_ITK	ITK_HUMAN	ITK	Q08881	355	620	266	620
400	45	TYR_JAK1−2	JAK1_HUMAN	JAK1	P23458	867	1154	288	1154
401	46	TYR_JAK2−2	JAK2_HUMAN	JAK2	O60674	841	1132	292	1132
402	47	TYR_JAK3−2	JAK3_HUMAN	JAK3	P52333	814	1105	292	1124
403	48	TYR_KDR	VGFR2_HUMAN	KDR	P35968	826	1170	345	1356
404	49	TYR_KIT	KIT_HUMAN	KIT	P10721	581	934	354	976
405	50	TYR_LCK	LCK_HUMAN	LCK	P06239	237	504	268	509
406	51	TYR_LMTK2	LMTK2_HUMAN	LMTK2	Q8IWU2	129	417	289	1503

bioRxiv preprint doi: https://doi.org/10.1101/2023.07.21.550125; this version posted September 3, 2023. The copyright holder for this preprint (which was not certified by peer review) is the author/funder, who has granted bioRxiv a license to display the preprint in perpetuity. It is made available under aCC-BY 4.0 International license.

407	52	TYR_LMTK3	LMTK3_HUMAN	LMTK3	Q96Q04	125	418	294	1460
408	53	TYR_LTK	LTK_HUMAN	LTK	P29376	502	787	286	864
409	54	TYR_LYN	LYN_HUMAN	LYN	P07948	239	507	269	512
410	55	TYR_MATK	MATK_HUMAN	MATK	P42679	227	488	262	507
411	56	TYR_MERTK	MERTK_HUMAN	MERTK	Q12866	579	864	286	999
412	57	TYR_MET	MET_HUMAN	MET	P08581	1068	1347	280	1390
413	58	TYR_MST1R	RON_HUMAN	MST1R	Q04912	1072	1351	280	1400
414	59	TYR_MUSK	MUSK_HUMAN	MUSK	O15146	567	866	300	869
415	60	TYR_NTRK1	NTRK1_HUMAN	NTRK1	P04629	502	791	290	796
416	61	TYR_NTRK2	NTRK2_HUMAN	NTRK2	Q16620	530	817	288	822
417	62	TYR_NTRK3	NTRK3_HUMAN	NTRK3	Q16288	530	834	305	839
418	63	TYR_PDGFRA	PGFRA_HUMAN	PDGFRA	P16234	585	960	376	1089
419	64	TYR_PDGFRB	PGFRB_HUMAN	PDGFRB	P09619	592	968	377	1106
420	65	TYR_PTK2	FAK1_HUMAN	PTK2	Q05397	414	686	273	1052
421	66	TYR_PTK2B	FAK2_HUMAN	PTK2B	Q14289	417	689	273	1009
422	67	TYR_PTK6	PTK6_HUMAN	PTK6	Q13882	183	451	269	451
423	68	TYR_RET	RET_HUMAN	RET	P07949	716	1015	300	1114
424	69	TYR_ROS1	ROS1_HUMAN	ROS1	P08922	1937	2225	289	2347
425	70	TYR_RYK	RYK_HUMAN	RYK	P34925	322	606	285	607
426	71	TYR_SRC	SRC_HUMAN	SRC	P12931	262	529	268	536
427	72	TYR_SRMS	SRMS_HUMAN	SRMS	Q9H3Y6	222	488	267	488
428	73	TYR_SYK	KSYK_HUMAN	SYK	P43405	362	635	274	635
429	74	TYR_TEC	TEC_HUMAN	TEC	P42680	362	629	268	631
430	75	TYR_TEK	TIE2_HUMAN	TEK	Q02763	816	1102	287	1124
431	76	TYR_TIE1	TIE1_HUMAN	TIE1	P35590	831	1117	287	1138
432	77	TYR_TNK1	TNK1_HUMAN	TNK1	Q13470	108	387	280	666
433	78	TYR_TNK2	ACK1_HUMAN	TNK2	Q07912	118	395	278	1038
434	79	TYR_TXK	TXK_HUMAN	TXK	P42681	263	527	265	527
435	80	TYR_TYK2-2	TYK2_HUMAN	TYK2	P29597	889	1179	291	1187
436	81	TYR_TYRO3	TYRO3_HUMAN	TYRO3	Q06418	510	796	287	890
437	82	TYR_YES1	YES_HUMAN	YES1	P07947	269	536	268	543
438	83	TYR_ZAP70	ZAP70_HUMAN	ZAP70	P43403	329	603	275	619

bioRxiv preprint doi: https://doi.org/10.1101/2023.07.21.550125; this version posted September 3, 2023. The copyright holder for this preprint (which was not certified by peer review) is the author/funder, who has granted bioRxiv a license to display the preprint in perpetuity. It is made available under aCC-BY 4.0 International license.

Supplementary Table 2. Pseudokinase domains in the human proteome

N	N (Fam)	Fam_Gene	SwissProt ID	Gene	Uniprot Acc.	Kinase Start	Kinase End	Kinase Length	Length protein
1	1	CAMK_CAMKV	CAMKV_HUMAN	CAMKV	Q8NCB2	16	294	279	501
2	2	CAMK_CASK	CSKP_HUMAN	CASK	O14936	4	284	281	926
3	3	CAMK_PLK5	PLK5_HUMAN	PLK5	Q496M5	1	74	74	336
4	4	CAMK_PSKH2	KPSH2_HUMAN	PSKH2	Q96QS6	55	328	274	385
5	5	CAMK_STK40	STK40_HUMAN	STK40	Q8N2I9	27	338	312	435
6	6	CAMK_TRIB1	TRIB1_HUMAN	TRIB1	Q96RU8	86	346	261	372
7	7	CAMK_TRIB2	TRIB2_HUMAN	TRIB2	Q92519	56	316	261	343
8	8	CAMK_TRIB3	TRIB3_HUMAN	TRIB3	Q96RU7	63	323	261	358
9	9	CAMK_TTN	TITIN_HUMAN	TTN	Q8WZ42	32170	32440	271	34350
10	1	CK1_VRK3	VRK3_HUMAN	VRK3	Q8IV63	158	463	306	474
11	1	OTHER_BUB1B	BUB1B_HUMAN	BUB1B	O60566	758	1029	272	1050
12	2	OTHER_EIF2AK4-1	E2AK4_HUMAN	EIF2AK4	Q9P2K8	272	547	276	1649
13	3	OTHER_MLKL	MLKL_HUMAN	MLKL	Q8NB16	193	471	279	471
14	4	OTHER_NRBP1	NRBP_HUMAN	NRBP1	Q9UHY1	56	335	280	535
15	5	OTHER_NRBP12	NRBP2_HUMAN	NRBP2	Q9NSY0	29	314	286	501
16	6	OTHER_PAN3	PAN3_HUMAN	PAN3	Q58A45	472	759	288	887
17	7	OTHER_PEAK1	PEAK1_HUMAN	PEAK1	Q9H792	1319	1673	355	1746
18	8	OTHER_PEAK3	PEAK3_HUMAN	PEAK3	Q6ZS72	163	405	243	473
19	9	OTHER_PIK3R4	PI3R4_HUMAN	PIK3R4	Q99570	18	321	304	1358
20	10	OTHER_POMK	SG196_HUMAN	POMK	Q9H5K3	73	341	269	350
21	11	OTHER_PRAG1	PRAG1_HUMAN	PRAG1	Q86YV5	984	1335	352	1406
22	12	OTHER_PXK	PXK_HUMAN	PXK	Q7Z7A4	138	404	267	578
23	13	OTHER_RNASEL	RN5A_HUMAN	RNASEL	Q05823	353	594	242	741
24	14	OTHER_RPS6KC1	KS6C1_HUMAN	RPS6KC1	Q96S38	332	1066	735	1066
25	15	OTHER_RPS6KL1	RPKL1_HUMAN	RPS6KL1	Q9Y6S9	145	547	403	549
26	16	OTHER_SCYL1	SCYL1_HUMAN	SCYL1	Q96KG9	6	271	266	808
27	17	OTHER_SCYL2	SCYL2_HUMAN	SCYL2	Q6P3W7	24	335	312	929
28	18	OTHER_SCYL3	PACE1_HUMAN	SCYL3	Q8IZE3	3	253	251	742
29	19	OTHER_STK31	STK31_HUMAN	STK31	Q9BXU1	702	980	279	1019
30	20	OTHER_STKLD1	STKL1_HUMAN	STKLD1	Q8NE28	20	305	286	680
31	21	OTHER_TBCK	TBCK_HUMAN	TBCK	Q8TEA7	1	281	281	893
32	22	OTHER_TEX14	TEX14_HUMAN	TEX14	Q8IWB6	219	520	302	1497
33	23	OTHER_ULK4	ULK4_HUMAN	ULK4	Q96C45	1	288	288	1275
34	1	RGC_GUCY2C	GUC2C_HUMAN	GUCY2C	P25092	468	767	300	1073
35	2	RGC_GUCY2D	GUC2D_HUMAN	GUCY2D	Q02846	502	815	314	1103
36	3	RGC_GUCY2F	GUC2F_HUMAN	GUCY2F	P51841	505	853	349	1108
37	4	RGC_NPR1	ANPRA_HUMAN	NPR1	P16066	507	829	323	1061
38	5	RGC_NPR2	ANPRB_HUMAN	NPR2	P20594	491	814	324	1047
39	1	STE_STRADA	STRAA_HUMAN	STRADA	Q7RTN6	61	387	327	431
40	2	STE_STRADB	STRAB_HUMAN	STRADB	Q9C0K7	50	377	328	418
41	1	TKL_ILK	ILK_HUMAN	ILK	Q13418	185	452	268	452
42	2	TKL_IRAK2	IRAK2_HUMAN	IRAK2	O43187	197	509	313	625
43	3	TKL_IRAK3	IRAK3_HUMAN	IRAK3	Q9Y616	152	453	302	596
44	4	TKL_KSR1	KSR1_HUMAN	KSR1	Q8IVT5	605	887	283	923
45	5	TKL_KSR2	KSR2_HUMAN	KSR2	Q6VAB6	658	938	281	950
46	1	TYR_EPHA10	EPHAA_HUMAN	EPHA10	Q5JZY3	637	910	274	1008
47	2	TYR_EPHB6	EPHB6_HUMAN	EPHB6	O15197	662	925	264	1021
48	3	TYR_ERBB3	ERBB3_HUMAN	ERBB3	P21860	701	975	275	1342
49	4	TYR_JAK1-1	JAK1_HUMAN	JAK1	P23458	575	855	281	1154
50	5	TYR_JAK2-1	JAK2_HUMAN	JAK2	O60674	537	815	279	1132
51	6	TYR_JAK3-1	JAK3_HUMAN	JAK3	P52333	513	787	275	1124
52	7	TYR_PTK7	PTK7_HUMAN	PTK7	Q13308	788	1070	283	1070
53	8	TYR_ROR1	ROR1_HUMAN	ROR1	Q01973	465	756	292	937
54	9	TYR_ROR2	ROR2_HUMAN	ROR2	Q01974	465	756	292	943
55	10	TYR_RYK	RYK_HUMAN	RYK	P34925	322	606	285	607
56	11	TYR_STYK1	STYK1_HUMAN	STYK1	Q6J9G0	105	390	286	422
57	12	TYR_TYK2-1	TYK2_HUMAN	TYK2	P29597	581	876	296	1187

Notes: Pseudokinase proteins may have catalytic activity that does not involve protein phosphorylation. The kinase domain in POMK is a protein O-mannose kinase. RNASEL is a 2'-5' endonuclease, in which the pseudokinase domain facilitates homodimerization. The pseudokinase domain of PAN3 participates in mRNA deadenylation. The RGC proteins contain active guanylyl cyclase domains; the kinase domains are inactive. PLK5 contains a truncated kinase domain at its N-terminus. The mouse ortholog contains a full kinase domain.
